# Researches on Hearing through the Medium of the Teeth and Cranial Bones

**Published:** 1880-05

**Authors:** Charles Hermon Thomas


### ARTICLE II.

### *Researches on Hearing through the Medium of the Teeth and Cranial Bones.*

BY CHARLES HERMON THOMAS, M. D.

Read before the Philadelphia County Medical Society, December 17, 1879.

[*Revised and Reprinted from the Philadelphia Medical Times, February* 28, 1879.]

In presenting the results of my investigations and experiments on the means and methods of transmitting vocal sounds to the internal ear by the way of the teeth and cranial bones, I shall but briefly describe the two instruments which have been exhibited here to-night, and which have received so large a measure of public attention during the past few weeks.

The audiphone resembles in form a Japanese fan, and is composed of vulcanite. When in use it is slightly bowed by cords connecting the handle and the upper edge, which edge is to be held in contact with the eye teeth of the hearer, the convex suface being turned towards the source of sound. The dentaphone is of the same model as a telephonic mouth-piece, its diaphragm being perforated at the centre for the attachment of a string, which serves to connect it with a small bit-piece, the latter to be held between the teeth of the hearer, who with one hand extends the mouth-piece with its opening towards and close to the face of the speaker, keeping the string in a state of tension.

It will be seen that the principles upon which their action is sounded are not new to us; indeed, are very generally known; but the remarkable results obtained by the application of these principles in the audiphone and dentaphone have fallen as a surprise alike upon the general public and the medical profession.

Hearing through the medium of the teeth and cranial bones is certainly not a novelty; on the contrary, the knowledge that sound may be thus conveyed has long been made use of for a variety of practical purposes. The application of the tuning-fork by aural surgeons in diagnosis is an instance familiar to all present. A patient of mine, whose hearing was much impaired in early life by scarlatina, informs me that in order to learn if his watch was running he has for many years been in the habit of placing it between his teeth, and of testing his clock also by applying his teeth to the mantel on which it stands, being otherwise unable to hear either of them. The same patient in his youth was accustomed to listen for the approach of a railroad train by placing his teeth upon the track; in this manner he was able to hear it some time before those who were about him, and who had normal hearing. A deaf friend enjoys music by placing one end of his cane against the piano and the other against his teeth. Another patient, an experienced engineer in charge of a stationary engine, has always practised listening to the sounds of its valves by placing one end of a stick upon the part nearest them and the other against his teeth, meanwhile stopping his ears with his thumbs, he having been taught this method as a part of his trade many years ago.

It has also been known that sound-waves produced in the air are communicated to contiguous bodies, causing them to vibrate in unison, though with varying degrees of intensity according to the density, elasticity, and form of such bodies. Furthermore, it was well known that vocal sounds were no exception to this rule, and that these, as well as others, could be transmitted, under suitable conditions, as longitudinal vibrations through long distances, as witness the familiar examples of the so-called "lovers' telegraph" and boys talking through a fence-rail. All this being known, it appears that a happy accident was all that was required to show that the complex vibrations of articulate speech as reproduced in solids might be conveyed through the teeth and cranial bones, and thus be rendered audible, as were the simpler sounds before mentioned; and, at the same time, to furnish a suitable means to accomplish the object.

My knowledge of the before-mentioned instruments and the results claimed for them led me to undertake a series of observations and experiments with a view (1) to demonstrate the principles upon which their action is founded; (2) to determine the practical value and range of use of these instruments; (3) to devise other and more convenient and less conspicuous forms of mechanism which might be substituted for them; (4) to improve the quality and increase the volume of the sound conveyed; (5) to discover new physiological and pathological facts relating to the functions of vocalization and hearing; and (6) to throw open to professional, and so to public, use the results gained, thus supplying data for further investigation and invention freed as far as possible from the restrictions which patents impose.

These researches involved the examination and oft-repeated re-examination of many deaf and partially deaf persons, the construction of new mechanism, and the testing, in different forms and under varied conditions, of a great variety of materials. In all this labor I had the valuable assistance of Dr. Edward D. Kirk, who will aid me in the repetition of several experiments before you to-night.

Before going farther, let me say that, though a number of novel results will here be shown, and some of them not without practical value, it is my opinion that osteophony as a subject worthy of study is not nearly exhausted, and that the future will develop other and far more useful applications of the method than have yet been reached.

The conclusions arrived at, briefly stated, are as follows:

1. As has been indicated, both the audiphone and dentaphone depend for their action upon the principles of acoustics that solids—in this case in the form of thin plates—vibrate in unison with the sound-waves produced in the air near them. In these instruments the vibrations are of sufficient force to be audible when conveyed to the internal ear through the medium of the teeth and cranial bones, independently of the ordinary channel of hearing,—the transmission being direct in the audiphone and indirect through the conducting string in the dentaphone.

Though, within certain limits, sound-vibrations in solids are readily conducted in this manner,—becoming audible in all cases where the internal ear is intact,—the relatively slight vibrations produced in dense substances by sound-waves propagated in the air near them are not recognizable as sound, except where the normal exercise of the function of hearing is suspended by disease of the middle ear or obstruction of the external portion of that organ. Normal hearing so far exceeds that obtained by the means in question as to render the comparatively smalll addition made through them inappreciable and as nothing in the presence of the greater sensation.

The side of the sound-receiving sheet governs the distance at which the instruments will act, and the dimensions must be increased in proportion as the source of sound is removed. The audiphone is, therefore, much better adapted than the dentaphone for use at a distance, the latter being only suited to transmit sounds emitted near its mouth-piece. The bowing strings of the former are not at all necessary to its perfect working, as its arched form may very conveniently be given to it by the hand, pressing its upper edge against the teeth. Not tension, but the arched form is the condition essential to its proper action, for this form is that best adapted to impart the impact of sound-waves against its convexity, which is then expended as a thrust of the arch against the teeth, these forming one of its abutments.

2. That these instruments are of great value in a considerable proportion of cases of deafness there is no reason to doubt, but there is no just ground for the public belief that with their aid the deaf are enabled to hear as well as those with ordinary hearing. On the contrary, they supply, I am convinced, but a very small fraction of normal hearing,—much *less* than a *hundredth part.* Neither instrument adds in the slightest perceptible degree to the hearing of those with perfect ears, except when the latter are securely stopped. I have repeatedly tested both instruments with this question in view, choosing as subjects those receiving from them decided benefit in listening to conversation and otherwise, and have as yet found no case in which a loud-ticking watch was heard when held close to the centre of the sound-receiver. Furthermore, when the watch was placed upon a large Koeing resonance box, thus multiplying the sound many times, and this placed so near to the diaphragm as barely to escape contact with it, still no sound was heard. Considering the law of acoustics, that the intensity of sound is inversely as the square of the distance of the sonorous body from the point at which it is heard, and taking into account the distance at which the sound described should be heard by the normal ear, my estimate of the strength, or rather the weakness, of the sound conveyed by these instruments will be seen to be justified. It is important that this be taken into account, for large numbers of the partially deaf suffer such disappointment at their failure to hear in full that they undervalue or altogether disregard a positive gain of many times their usual hearing. The difference between normal hearing and that derived through these means is hardly less marked than that between sunlight and candle-light; nevertheless, this very small fraction is of priceless value in many cases, for to those who practically hear nothing without them, who sit in acoustic darkness, the gain is all the difference between nothing and something,—scarcely less than infinity.

In view of certain strongly expressed statements which have obtained currency, the results to be derived from the use of the audiphone in deaf-muteism are likely to prove very disappointing. Repeated tests show that those who are able to hear with the aid of the audiphone hear *their own voices* perfectly without it: while those who are unable to hear their own voices without it can hear neither their own nor any other voice with it. It is, therefore, worthless to those who do not possess the faculty of self-hearing.

Practice is required in using either instrument, especially in cases where hearing has long been abolished, and the suggestion which has been made, to read aloud to the patient while he follows the printed words with the eyes, is a good one. Besides this, practice with the watch or tuning-fork against the teeth, thus directing the attention to receiving the sound through the new channel, will prove valuable as an educator of the disused sense. The rod osteophone—hereafter to be described—has a special value for the same purpose.

This class of instruments should be in the hands and under the special direction of physicians, among other reasons, because caution is necessary to prevent their being relied on to the neglect of proper examination and treatment of the ears. Probably the most striking results to be found from their use would be in obstruction of the external ear by impacted cerumen; but manifestly it would be malpractice to make use of such means instead of effecting the removal of the offending material.

It is perhaps too soon to decide the relative merits of the ear-trumpet and the osteophone, but enough is known to render it probable that the osteophone will either substitute or supplement the former in many instances. And, certainly, it is far more agreeable for most persons to talk towards the surface of a curved sheet than into the cavity of a trumpet. Moreover, the distress and injury to the ears sometimes caused by trumpets are avoided altogether in the osteophones. As to artificial teeth, they limit to a great degree the value of most osteophones.

3. The audiphone is open to the objection that during its use it obscures to a certain extent the features of the user, and the dentaphone is held more or less in the line of vision. Both instruments are open to the still more serious objection that they each require the constant service of at least one hand during their use. With neither can the user hear when his hands are engaged in other employment. To remedy these defects I have made the following device.

A larg receiving diaphragm is attached, in an arched form, to a rod of wood or metal. The rod is bent in the form of a pipe stem, one end ot which is to be held firmly between the teeth as a pipe is held, thus enabling the uses to listen to sounds about him, and, at the same time, leaving his hands free for other occupation. The diaphragm being below and away from the face, it is comparatively inconspicuous.

Ornamental fans of almost all sorts may be utilized for occasional use, and, when coated with shellac and tipped with ivory or hard rubber, may be made to answer fairly well, though they will prove unsatisfactory for ordinary uses. Many other forms of instruments have been found to answer the purpose of conveying vocal vibrations: thus, a piece of yellow pine wood, turned out into trumpet-shape, about two feet long, with an expanded end three inches in diameter, may be used for conversation, the small end being applied to the teeth of the deaf person and the opposite end held close to the mouth of the speaker. In this manner a very good volume of sound is conveyed. A tense string connecting the upper teeth of the two about to converse also answers the same purpose.

4. The tone of the voice differs materially when conveyed by different substances. Where these are lacking in resonance (as in celluloid and binders' board,) flatness is the result, while others which are over-resonant or over-persistent in their vibrations (as vulcanite and ferrotype metal) yield ringing or confused sounds. The quality needed is that possessed by good sounding-boards, of instantly responding to contiguous sounds and maintaining them during their continuance, and also of instantly ceasing to vibrate upon the cessation of the causative sound. This right sort of elasticity of resonance, that capable of reproducing human voice-tones in their purity, is possessed to a high degree by fullers' board (or press-board,) which, when treated with shellac varnish and thoroughly dried, has proved not only far better than other paper or cardboards, but also a great improvement upon the sheet metals or hard rubber; lacking the "reverberations" and "roaring sounds" of the latter, as they are described by different patients upon whom they have been tested. Besides, owing to its greater flexibility, it is less destructible than either these or the thin sheets of wood which otherwise answered the purpose, while its cost is comparatively trivial.

A powerful osteophone, one that excels by many times in the volume of sound transmitted either audiphone or dentaphone, consists simply of a small rod of hard wood,—a convenient size being about two feet long and a quarter of an inch thick,—one end of which is placed against the teeth of the speaker, the other resting against or between the teeth of the person hard of hearing. If the speaker now articulates in a natural tone of voice, the vocal vibrations will be transmitted in great volume through the teeth and thence to the ears of the deaf person. It will also convey the voice distinctly when placed against the forehead or other portions of the skull of the hearer, and will convey perfectly audible speech from the skull of one to that of the other. Again, instead of the speaker holding it against his teeth, he may place it against the upper part of his chest, when, upon using his voice, the sound will be conveyed as before.

5. A physiological fact, not duly recognized or appreciated hitherto, so far as I am aware, is that sensible vibrations, produced by and corresponding to those of the voice, are propagated in the hard palate and base of the skull of persons speaking in ordinary tones. These vibrations are found to be transmitted throughout the skull with varying degrees of intensity, according to the point of observation chosen. In some localities—as the forehead, the front teeth of the upper jaw, and the top of the head—which are specially vibratory, these are so powerful as to be readily felt by the hand of the observer pressed upon these parts of another person during the act of speech. They may also be evidenced as articulate sounds, and distinctly heard as such, by any one, through the use of the rod-osteophone, for, as has been observed, by its means the volume of sound consciously received is a perceptible addition to ordinary hearing. Through their agency the voice may be conveyed directly from the skull of the speaker to that of the deaf listener by simply bringing the heads themselves in contact. The existence of these vibrations accounts for the further fact, before mentioned and which I have taken much pains to verify, that in certain cases of extreme deafness the patient hears his own voice with the utmost distinctness, even though speaking in low tones, when like sounds produced in his very ears would be totally inaudible. It seems evident, then, that auto-audition for deaf and well alike includes among its leading factors the bone-vibrations spoken of; and the topographical relations of the larynx and the vault of the pharynx to that portion of the temporal bone which includes the internal ear supply good *a priori* grounds, if any such were needed, for reaching the same conclusion.

---

